# Slide positivity, trends, and risk factors of febrile *Plasmodium vivax* malaria along the Thailand-Myanmar border, 2018–2023

**DOI:** 10.1186/s40249-025-01350-4

**Published:** 2025-08-06

**Authors:** Pyae Linn Aung, Nattawan Rachaphaew, Piyarat Sripoorote, Khaing Zin Zin Htwe, Kritsana Suk-aum, Jaranit Kaewkungwal, Saranath Lawpoolsri, Liwang Cui, Jetsumon Sattabongkot

**Affiliations:** 1 Mahidol Vivax Research Unit, Faculty of Tropical Medicine, Mahidol University, Bangkok, Thailand; 2 Center of Vector Borne Disease Control 2.3, Ministry of Public Health, Tak, Thailand; 3 Department of Tropical Hygiene, Faculty of Tropical Medicine, Mahidol University, Bangkok, Thailand; 4 Division of Infectious Diseases and International Medicine, Department of Internal Medicine, Morsani College of Medicine, University of South Florida, 3720 Spectrum Boulevard, Suite 304, Tampa, FL 33612 USA

**Keywords:** Malaria, *Plasmodium vivax*, Risk factor, Slide positivity, Thailand, Trend

## Abstract

**Background:**

*Plasmodium vivax* is the predominant malaria species in many Southeast Asian countries. Eliminating all human malaria species by 2030 requires greater focus on *P. vivax*, with targeted measures to address its unique challenges. This study evaluated slide positivity rates, temporal trends, and risk factors associated with febrile *P. vivax* infections in a malaria-endemic district along the Thailand-Myanmar border.

**Methods:**

This study employed a community-based longitudinal surveillance design over six years (January 2018–December 2023). Data were collected through routine passive case detection at field malaria clinics using extended, standardized case record forms. Malaria diagnosis was conducted via microscopy examination. Descriptive statistics and logistic regression models were used for data analysis.

**Results:**

Among 13,347 febrile malaria-suspected patients, the cumulative slide positivity rate for *P. vivax* was 11.0%. Although no distinct seasonal peaks were observed, *P. vivax* cases generally increased in April and again in November and December. Multivariable logistic regression analysis identified several significant risk factors for febrile *P. vivax* infection, including school-aged children (5–14 years) (a*OR*: 1.56, 95% *CI:* 1.24–1.97), working-age adults (15–34 years) (a*OR*: 1.43, 95% *CI:* 1.02–2.00), males (a*OR*: 1.19, 95% *CI:* 1.06–1.35), Myanmar nationals (a*OR*: 2.37, 95% *CI:* 2.01–2.80), and other non-Thai nationals, such as individuals from Laos and Cambodia (a*OR*: 5.50, 95% *CI:* 3.36–8.90). A history of malaria (a*OR*: 1.59, 95% *CI:* 1.38–1.83), recent travel within two weeks (a*OR*: 2.38, 95% *CI:* 1.94–2.92), and engagement in livestock-related occupations (a*OR*: 2.49, 95% *CI:* 1.14–5.35) were also associated with higher odds of infection. In contrast, being unemployed (a*OR*: 0.55, 95% *CI:* 0.36–0.81), working in occupations such as maid, driver, or teacher (a*OR*: 0.78, 95% *CI:* 0.66–0.93), and consistent use of bed nets (a*OR*: 0.39, 95% *CI:* 0.30–0.51) significantly reduced infection risk.

**Conclusions:**

This study identified a relatively high slide positivity rate of febrile *P. vivax* infection in a malaria-endemic district in western Thailand along the Myanmar border. Strengthening malaria surveillance, targeting high-risk populations, ensuring treatment adherence, and promoting early care-seeking behavior are crucial for reducing *P. vivax* transmission and advancing malaria elimination efforts.

**Graphical abstract:**

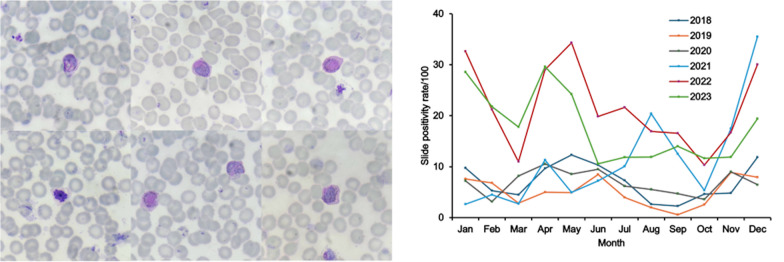

## Background

Malaria remains a significant global public health concern. Although substantial progress has been made in reducing the disease burden, recent trends indicate a resurgence. According to the latest World Malaria Report [1], approximately 263 million malaria cases were reported globally in 2023, marking an increase of 11 million cases compared to the previous year. Southeast Asia (SEA) is the second most malaria-affected region after Africa, with ten countries contributing to the regional burden. Among major malaria species, most SEA countries, except India, Indonesia, Myanmar, and Bangladesh, have reported either a predominance of *Plasmodium vivax* or an increasing trend compared to *P. falciparum*. Similarly, in the Greater Mekong Subregion (GMS), most countries have observed a rising trend of *P. vivax*, except that China successfully eliminated malaria in June 2021 [1, 2]. Thailand has made substantial progress in malaria control, particularly between 2015 and 2021. However, a recent resurgence has been reported, with over 16,000 malaria cases recorded in 2023, representing an almost five-fold increase compared to 2021 [1]. Border malaria remains a major challenge, with transmission concentrated along the Thailand-Myanmar border, particularly following the 2021 military coup in Myanmar, which led to large-scale population displacements into Thailand. In 2024, more than 55% of reported malaria cases in Thailand were detected among short- or long-term Myanmar migrants. Among the 2023 malaria cases, *P. vivax* accounted for 94%, a substantial increase from 49% over the past decade [3]. Despite this resurgence, Thailand aims to eliminate malaria nationwide by 2030. However, eliminating *P. vivax* is more challenging than *P. falciparum*, primarily due to the persistence of dormant liver-stage hypnozoites [4, 5]. Therefore, targeted interventions against *P. vivax* are essential to accelerate malaria elimination efforts.

For malaria surveillance, particularly in endemic areas, Thailand has established malaria clinics across the country since 1965 [6]. Through this network, the country implements community-based intensive malaria elimination strategies targeting *P. vivax*. These strategies include early diagnosis and treatment, glucose-6-phosphate dehydrogenase (G6PD) testing before administering the 14-day primaquine (PQ) regimen, and reactive case detection as part of the 1-3-7 strategy to identify potential localized transmission [7]. In addition, vector control measures are implemented to reduce transmission risk. As part of its core vector control strategy, Thailand conducts mass distribution of long-lasting insecticide-treated nets (LLINs), followed by top-up distributions for high-risk populations such as pregnant women and forest-goers [8]. However, the proper use of LLINs and other bed nets remains suboptimal, particularly among forest-related populations [9, 10]. Furthermore, Thailand has not yet implemented Directly Observed Therapy (DOT) for the 14-day PQ regimen, although follow-up visits after treatment completion are encouraged. As a result, treatment adherence remains a challenge, leading to relapses of *P. vivax* and hindering elimination efforts.

In Thailand, major malaria vectors, including *Anopheles minimus*, *An. dirus*, *An. annularis* and *An. maculatus*, are widely reported [11, 12]. Generally, after acquiring malaria through the bite of an infected mosquito, individuals develop a fever with chills and rigor following the incubation period. For *P. vivax*, the incubation period typically ranges from 10 to 17 days [13]. However, in individuals residing in malaria-endemic areas for extended periods, the severity of symptoms may vary depending on the degree of naturally acquired immunity. Some individuals may remain asymptomatic while carrying parasites at low densities, whereas others, particularly those from low-endemic regions or those with high parasite density, are more likely to present with clinical symptoms [14–16]. Among these two groups, the potential for onward malaria transmission differs, with symptomatic individuals contributing more significantly to transmission [17]. Therefore, an effective surveillance system and immediate treatment of symptomatic malaria cases are critical to interrupt localized transmission and achieve malaria elimination.

With *P. vivax* becoming predominant in many SEA countries and persistent challenges in its elimination, a literature review conducted on PubMed in February 2025 found limited studies summarizing risk factors associated with *P. vivax* infections. A study from Ethiopia reported higher infection risks among children under five years and school-aged children [18]. Another study identified an increased risk among travelers visiting endemic areas [19]. Additionally, a study conducted among ethnic populations in Myanmar found that males under 15 years of age were more likely to present clinical symptoms compared to females [20]. Given the scarcity of available literature, this study aims to evaluate slide positivity rates, seasonal trends, and risk factors associated with febrile *P. vivax* infections in one of Thailand’s malaria-endemic areas with a high burden of *P. vivax* malaria.

## Methods

### Study design

This study employed a community-based longitudinal surveillance design to investigate malaria epidemiology. Data were collected over six years, from January 2018 to December 2023.

### Study location

**Fig. 1:**
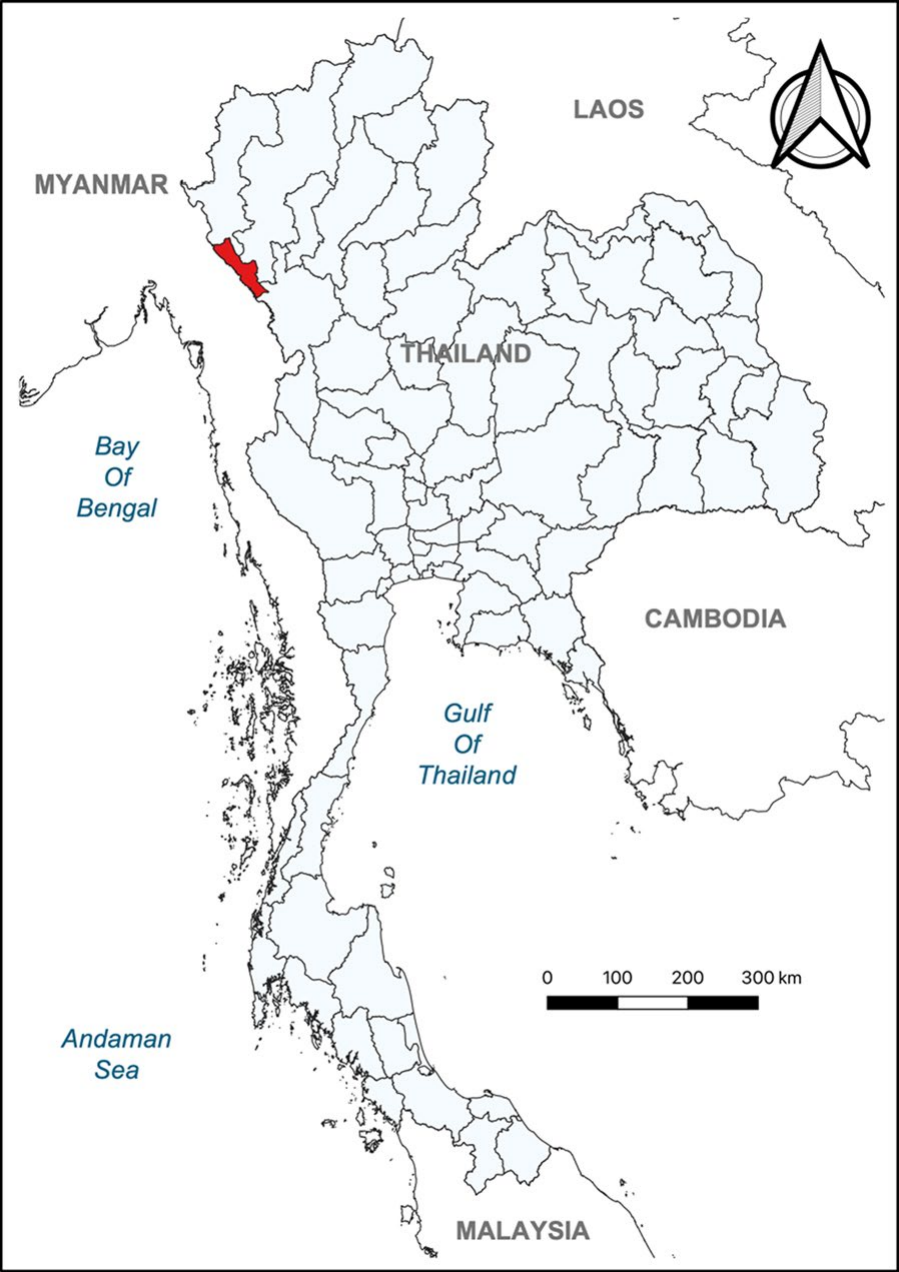
Geographic location of Tha Song Yang District (highlighted in red) in Tak Province, western Thailand, along the border with Myanmar. The base map shapefiles were obtained from the DIVA-GIS database (https://diva-gis.org) and modified using QGIS for Mac (version 3.34.2-Prizren)

Malaria remains endemic in Thailand, with the highest prevalence occurring in the western provinces bordering Myanmar. This study specifically focused on Tak Province, which has consistently reported the highest number of malaria cases. In 2024, Tak Province accounted for more than 7300 malaria cases, representing over 48% of the total reported cases in Thailand [3]. Among the nine districts in Tak Province, Tha Song Yang was purposively selected as the study site due to its high malaria prevalence (Fig. 1). In 2024, Tha Song Yang district contributed approximately 28% of the total malaria cases in Tak Province [3]. The district has a population of approximately 100,000 and is situated along the Moei River, which forms the natural border with Myanmar. Cross-border movement is frequent, as individuals can easily travel between the two countries by boat or on foot, particularly during the dry season.

In Thailand, including in Tha Song Yang district, malaria diagnosis and treatment are free, regardless of an individual’s citizenship or migration status. These services are primarily delivered through malaria clinics, which have been operational since 1965 [6]. In Tha Song Yang district, five malaria clinics serve the community. Based on feasibility, logistics, and reported malaria incidence, three clinics and one malaria post were purposively selected for the study. Each clinic oversees malaria-related activities for approximately 10–15 villages and operates during regular working hours, except on public holidays. On average, three to five staff members are stationed at each clinic, including basic healthcare workers, a data officer, and a trained microscopist.

### Data collection

Malaria clinics conduct passive case detection (PCD) as part of routine malaria surveillance. Individuals presenting with malaria-like symptoms or seeking malaria testing can receive free consultations. Clinic staff performs basic clinical assessments and malaria microscopy by preparing thin and thick blood films. Based on test results, appropriate treatment is provided according to national guidelines. For confirmed *P. vivax* cases, patients receive chloroquine at a total dose of 25 mg/kg over three days. Those with normal G6PD status are also prescribed primaquine at 0.25 mg/kg daily for 14 days to achieve radical cure and prevent relapse [21].

For this longitudinal study, a case record form (CRF) was developed in Thai, incorporating elements from previous malaria epidemiological studies. The CRF was validated by malaria experts and consists of five sections: basic demographic information, present illness history, malaria diagnosis and treatment, malaria history, and recent travel history. Demographic data included age, sex, nationality, occupation, and education level. Present illness history included measured body temperature and self-reported fever history. The malaria diagnosis and treatment section recorded diagnostic results and prescribed treatment. The malaria history section documented previous malaria infections, while the final section recorded travel within the past two weeks before diagnosis.

Before initiating data collection, all malaria clinic staff underwent a one-day training session on CRF data collection and informed consent procedures. The designated data focal person at each clinic completed the CRFs for suspected malaria patients undergoing blood examination. Completed CRFs were scanned and uploaded into the data reporting system. The data were managed using DF-Discovery software, a cloud-based database hosted on Amazon Web Services. The system displayed scanned CRFs alongside a pre-designed digital version, into which data were manually entered. A field data team performed monthly quality control checks to ensure accuracy. Additionally, the central data team cross-checked incoming data and regularly communicated with field staff to resolve errors, discrepancies, or missing entries. The finalized dataset was retrieved from the system for the period January 2018 to December 2023. All data were translated into English by the research team, including native Thai scientists.

### Malaria diagnosis

Malaria microscopy was performed at malaria clinics by trained field microscopists, who completed initial malaria microscopy training and periodic refresher courses. Thick and thin blood films were prepared, stained with 10% Giemsa, and examined under a light microscope at 1000 × magnification following the WHO standard protocol [22]. To ensure diagnostic accuracy, prepared slides were dried, stored in slide boxes, and transported bi-weekly or monthly to the Mahidol Vivax Research Unit, Faculty of Tropical Medicine, Mahidol University, for expert confirmation. An experienced microscopist re-examined all slides, and the results were recorded in the database. Only these expert-confirmed results were used in the final analysis. This dual-reading process served as an internal quality control mechanism. Although microscopy is the gold standard for malaria diagnosis in field settings, low-density parasitemia may still be missed. However, as this study relied on febrile patients, who typically present with relatively higher parasite densities, the likelihood of missed *P. vivax* infections was reduced.

### Sample selection and data processing

The case-based surveillance dataset was initially prepared in Microsoft Excel, version 16.97.2 (25,052,611), with missing data reviewed and verified by the research team. Over the six years, the dataset contained 14,213 records. Fever status was determined based on two criteria: a measured body temperature of ≥ 38 °C at the malaria clinic or a self-reported fever within the three days preceding clinic consultation. As this study focused on febrile *P. vivax* malaria cases, certain records were excluded. These included 809 records of non-febrile patients, three with missing fever status, and eight with repeated tests or infections. Repeat entries were identified using assigned patient IDs and cross-checked against sociodemographic characteristics such as age and sex to ensure accurate deduplication. While some of these cases may have represented relapses rather than new infections, they were not distinguished in the present study. To avoid overestimation of risk, only the most recent febrile episode was retained for individuals with multiple infections. Additionally, 46 records with extensive missing data were excluded. After these exclusions, the final dataset included 13,347 records for analysis.

### Data analysis

Data analysis was conducted using R software (version 4.4.2 for Mac; R foundation for Statistical Computing, Vienna, Austria) and comprised four components. First, slide positivity rates (SPR) for all malaria species were calculated for each year, as well as cumulatively over the six years, by dividing the total number of malaria cases by the total number of samples examined. Second, trends in monthly SPR for *P. vivax* cases were analyzed and visualized using line graphs. Third, demographic characteristics of the study population were summarized using frequencies and percentages. Finally, univariate and multivariate logistic regression models were used to assess factors associated with febrile *P. vivax* infection. All variables included in the unadjusted model were retained in the multivariable model to identify true associations. Crude odds ratios (c*OR*) and adjusted odds ratios (a*OR*) with 95% confidence intervals (*CI*s) were reported. Statistical significance was determined based on whether the 95% *CI* included the null value (1.0). Prior to multivariable analysis, multicollinearity among independent variables was assessed using generalized variance inflation factors (GVIF). All adjusted GVIF values were below 1.5, indicating no significant collinearity among the key variables. Therefore, all variables were retained in the final model.

## Results

### Slide positivity rates

Between 2018 and 2023, 13,347 febrile patients were examined for malaria using microscopy. The number of participants tested each year ranged from a minimum of 2052 to a maximum of 2910, except in 2021 when only 955 individuals were tested due to the impact of the COVID-19 pandemic and its associated preventive measures. The cumulative SPR for all species over the study period was 11.6%, with annual fluctuations. Notably, SPR increased in recent years, particularly after 2021, with rates of 10.5% in 2021, 21.2% in 2022, and 17.4% in 2023. Among the 1541 confirmed malaria cases, *P. vivax* accounted for the vast majority (1472 cases, 95.5%), followed by *P. falciparum* (50 cases, 3.2%), *P. malariae* (16 cases, 1.0%), and mixed infections (3 cases, 0.2%). The cumulative SPR for *P. vivax* throughout the study period was 11.0% (Table 1).

**Table 1:** Slide positivity rates of febrile malaria cases diagnosed by microscopy in Tha Song Yang District, Tak Province, Thailand, from 2018 to 2023

Year	Total slides examined	Malaria infections					
		*Plasmodium vivax*	*P. falciparum*	*P. malariae*	Mixed infections	Total	SPR (%)
2018	2910	201	24	0	1	226	7.8
2019	2568	124	11	0	0	135	5.3
2020	2052	143	1	0	1	145	7.1
2021	955	100	0	0	0	100	10.5
2022	2338	486	7	3	0	496	21.2
2023	2524	418	7	13	1	439	17.4
Total	**13,347**	**1472**	**50**	**16**	**3**	**1541**	**11.6**

*SPR* Slide positivity rate/ 100; *Mixed infection* Coinfections involving *P. falciparum* and other malaria species; Bold values indicate totals

### Trends of febrile P. vivax malaria

**Fig. 2:**
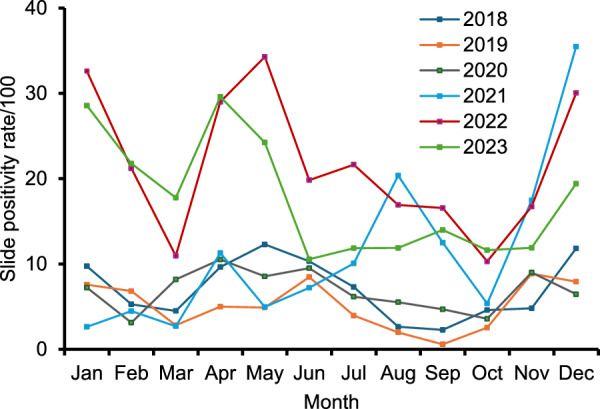
Monthly slide positivity rate (SPR) of febrile *Plasmodium vivax* malaria cases detected through passive case detection in Tha Song Yang District, Tak Province, Thailand, from January 2018 to December 2023

Among the 1472 *P. vivax* cases, the monthly SPR for each year was illustrated in Fig. 2 to assess seasonal variations. Overall, no distinct seasonal peaks in febrile *P. vivax* malaria cases were observed. However, a general increase in cases was noted in April, followed by another rise in November and December. The highest peak (29.0%) occurred in April 2022, whereas the lowest recorded SPR (2.3%) was in September 2018.

### Risk factors associated with febrile P. vivax malaria

A total of 13,278 individuals were tested for malaria using microscopy, excluding those with non-*P. vivax* infections. The majority of study participants were Thai citizens (89.2%) and children aged 5–14 years (32.2%). Most were male (57.6%), students or children (48.9%), and illiterate (43.3%). More than half (61.3%) had no prior history of malaria, and the majority (95.5%) reported no travel history in the two weeks before testing. Additionally, 76.3% reported using bed nets every night (Table 2).

**Table 2:** Sociodemographic, clinical, and behavioral characteristics of febrile patients tested for malaria by microscopy in Tha Song Yang District, Thailand (*n* = 13,278)

Characteristics	Number	Percentage (%)
Age (years)		
< 5	1734	13.1
5–14	4271	32.2
15–34	3913	29.4
≥ 35	3360	25.3
Sex		
Female	5624	42.4
Male	7654	57.6
Nationality		
Thai	11,845	89.2
Myanmar	1356	10.2
Other (Laos, Cambodia)	77	0.6
Occupation		
Agriculture/Farmer	3356	25.3
Child/Student	6495	48.9
Government officer	110	0.8
Labor	82	0.6
Livestock	32	0.2
Merchant	196	1.5
Monk	43	0.3
Soldier/Police	88	0.7
Unemployed	526	4.0
Other (maid, driver, or teacher)	2350	17.7
Education		
College/University and above	227	1.7
Secondary school	1758	13.2
Primary school	5538	41.8
Illiterate	5755	43.3
History of malaria		
No	8137	61.2
Yes	2703	20.4
Do not remember	2438	18.4
Travel history in the last two weeks		
No	12,683	95.5
Yes	595	4.5
Bed net usage		
Never	411	3.1
< 1 day per week	155	1.2
1–3 days per week	557	4.2
4–6 days per week	2018	15.2
Every day	10,137	76.3

Among the 1472 *P. vivax*-positive individuals, most were aged 15–34 years (527 cases, 35.8%) and male (969 cases, 65.8%). The majority were Thai nationals (1148 cases, 78.0%) and unemployed children or students (686 cases, 46.6%). Nearly half (694 cases, 47.1%) were illiterate, and more than half (773 cases, 52.5%) had no previous malaria history. Additionally, 1307 cases (88.8%) had no travel history within the past two weeks, and 824 cases (56.0%) reported using bed nets daily (Table 3).

**Table 3:** Unadjusted and adjusted odds ratios for factors associated with febrile *Plasmodium vivax* malaria among patients tested in Tha Song Yang District, Thailand, 2018–2023

Characteristics	*P. vivax* infection		c*OR* (95% *CI*)	a*OR* (95% *CI*)
	Positive (*n* = 1472)	Negative (*n* = 11,806)		
	*n* (%)	*n* (%)		
Age (years)				
< 5	126 (7.3)	1608 (92.7)	Reference	Reference
5–14	493 (11.5)	3778 (88.5)	1.67 (1.36–2.05)	1.56 (1.24–1.97)
15–34	527 (13.5)	3386 (86.5)	1.99 (1.63–2.44)	1.43 (1.02–2.00)
≥ 35	326 (9.7)	3034 (90.3)	1.37 (1.11–1.70)	1.05 (0.74–1.49)
Sex				
Female	503 (8.9)	5121 (91.1)	Reference	Reference
Male	969 (12.7)	6685 (87.3)	1.48 (1.32–1.65)	1.19 (1.06–1.35)
Nationality				
Thai	1148 (9.7)	10,697 (90.3)	Reference	Reference
Myanmar	292 (21.5)	1064 (78.5)	2.56 (2.21–2.95)	2.37 (2.01–2.80)
Other (Laos, Cambodia)	32 (41.6)	45 (58.4)	6.63 (4.16–10.4)	5.50 (3.36–8.90)
Occupation				
Agriculture/Farmer	412 (12.3)	2944 (87.7)	Reference	Reference
Child/Student	686 (10.6)	5809 (89.4)	0.84 (0.74–0.96)	1.16 (0.87–1.53)
Government officer	3 (2.7)	107 (97.3)	0.20 (0.05–0.53)	0.28 (0.06–0.91)
Labor	14 (17.1)	68 (82.9)	1.47 (0.79–2.56)	1.43 (0.75–2.58)
Livestock	14 (43.8)	18 (56.2)	5.56 (2.70–11.2)	2.49 (1.14–5.35)
Merchant	12 (6.1)	184 (93.9)	0.47 (0.24–0.81)	0.60 (0.31–1.05)
Monk	7 (16.3)	36 (83.7)	1.39 (0.56–2.96)	1.32 (0.52–2.89)
Soldier/Police	12 (13.6)	76 (86.4)	1.13 (0.58–2.01)	0.92 (0.45–1.72)
Unemployed	29 (5.5)	497 (94.5)	0.42 (0.28–0.60)	0.55 (0.36–0.81)
Other (maid, driver, or teacher)	283 (12.0)	2067 (88.0)	0.98 (0.83–1.15)	0.78 (0.66–0.93)
Education				
College/University and above	12 (5.3)	215 (94.7)	Reference	Reference
Secondary school	213 (12.1)	1545 (87.9)	2.47 (1.42–4.74)	1.38 (0.73–2.87)
Primary school	553 (10.0)	4985 (90.0)	1.99 (1.15–3.78)	1.07 (0.56–2.23)
Illiterate	694 (12.1)	5061 (87.9)	2.46 (1.43–4.67)	1.26 (0.66–2.62)
History of malaria in the last year				
No	773 (9.5)	7364 (90.5)	Reference	Reference
Yes	402 (14.9)	2301 (85.1)	1.66 (1.46–1.89)	1.59 (1.38–1.83)
Do not remember	297 (12.2)	2141 (87.8)	1.32 (1.14–1.52)	1.28 (1.10–1.49)
Travel history in the last two weeks				
No	1307 (10.3)	11,376 (89.7)	Reference	Reference
Yes	165 (27.7)	430 (72.3)	3.34 (2.76–4.02)	2.38 (1.94–2.92)
Bed net usage				
Never	99 (24.1)	312 (75.9)	Reference	Reference
< 1 day per week	44 (28.4)	111 (71.6)	1.25 (0.82–1.89)	1.33 (0.86–2.04)
1–3 days per week	125 (22.4)	432 (77.6)	0.91 (0.68–1.23)	0.97 (0.71–1.33)
4–6 days per week	380 (18.8)	1638 (81.2)	0.73 (0.57–0.94)	0.83 (0.64–1.09)
Every day	824 (8.1)	9313 (91.9)	0.28 (0.22–0.35)	0.39 (0.30–0.51)

*cOR* crude odds ratio; *aOR* adjusted odds ratio; *95% CI* 95% confidence interval

Multivariable logistic regression analysis identified several significant risk factors for febrile *P. vivax* infection (Table 3). School-aged children (5–14 years) had significantly higher odds of infection than children under 5 years (a*OR*: 1.56, 95% *CI:* 1.24–1.97). Similarly, working-age adults (15–34 years) had an increased risk (a*OR*: 1.43, 95% *CI:* 1.02–2.00). Males were at higher risk than females (a*OR*: 1.19, 95% *CI:* 1.06–1.35). Myanmar nationals (a*OR*: 2.37, 95% *CI:* 2.01–2.80) and other non-Thai nationals (e.g., individuals from Laos and Cambodia) (a*OR*: 5.50, 95% *CI:* 3.36–8.90) had significantly higher odds of infection than Thai nationals. Individuals with a history of malaria had a greater likelihood of infection (a*OR*: 1.59, 95% *CI:* 1.38–1.83). Those who had traveled in the two weeks preceding the survey were also at higher risk (a*OR*: 2.38, 95% *CI:* 1.94–2.92). Occupation was a significant predictor: individuals engaged in livestock-related activities had higher odds of infection than those in agriculture (a*OR*: 2.49, 95% *CI:* 1.14–5.35). In contrast, being unemployed (a*OR*: 0.55, 95% *CI:* 0.36–0.81) or classified under other occupations, such as maid, driver, or teacher (a*OR*: 0.78, 95% *CI:* 0.66–0.93), appeared to be protective factors against *P. vivax* infection. Consistent use of bed nets significantly reduced infection risk, with individuals who reported daily bed net use having lower odds of *P. vivax* infection than those with irregular use (a*OR*: 0.39, 95% *CI:* 0.30–0.51).

## Discussion

In Thailand and other GMS countries, *P. vivax* is the predominant malaria species [1]. In this study, more than 95% of reported malaria cases were due to *P. vivax*, aligning with national data indicating that *P. vivax* accounted for 98% of reported malaria cases in 2023 [1, 3]. Several studies have demonstrated a strong association between seasonal variation and malaria transmission trends [23–25]. Malaria incidence typically peaks during the pre-monsoon and monsoon seasons, as increased rainfall creates favorable conditions for vector breeding. However, in border regions with high malaria receptivity, characterized by the presence of competent malaria vectors and frequent population movements among vulnerable groups, malaria transmission can occur year-round, with occasional unexpected surges. Additionally, climate change has altered seasonal transmission patterns, as unpredictable rainfall and rising temperatures extend mosquito longevity and enhance breeding site availability [26, 27].

The use of malaria prevention measures for young children largely depends on caregivers. In general, parents and adult caregivers prioritize the care of young children, including ensuring bed net use [28]. Consequently, in this study, school-aged children and working-age adults had a higher risk of *P. vivax* infection than young children. School-going children may be exposed to infection at school or playgrounds before bedtime, often outside peak *Anopheles* biting hours [20, 29]. Similarly, working-age adults, particularly those engaged in night shifts, may face challenges in using preventive measures such as bed nets [10]. In many Asia cultures, men are often responsible for earning household income, while women typically manage household affairs, including childcare. Additionally, community-based health promotion activities and malaria interventions are usually conducted during the daytime, when participation is higher among women and the elderly. As a result, men tend to have lower engagement in malaria prevention practices, increasing their risk of infection due to occupational and outdoor exposures in vector-abundant areas [20, 26]. Therefore, school-based malaria control programs, along with targeted interventions for working-aged men, could be effective in reducing malaria transmission in these populations.

The study areas are located along the Myanmar-Thailand border, separated by a river. However, individuals can easily cross between the two countries without passing through official border checkpoints, either by boat or on foot during the dry season. Notably, following the political unrest and military coup in Myanmar in 2021, there was a significant increase in cross-border migration. This surge in population movement coincided with an increase in malaria cases, as reflected in both national surveillance data and the findings of this study [3]. A recent study also indicated that Myanmar migrants in Thailand have limited access to malaria services, making them more vulnerable to infection [30]. Consistently, our study found a higher risk of *P. vivax* infection among non-Thai citizens, particularly those from Myanmar. Similarly, individuals from other low-endemic countries, such as Laos and Cambodia, also exhibited a higher risk of malaria. People from low-endemicity regions may have lower immunity, making them more susceptible to infection when entering high-transmission areas [31]. Conversely, many Thai nationals invest in and cultivate agricultural land on the Myanmar side of the border, where land and labor costs are lower than in Thailand. As a result, frequent cross-border movement is common in these study areas. Accordingly, individuals who had traveled in the two weeks before the survey had a significantly higher risk of *P. vivax* infection [23]. To mitigate cross-border malaria transmission, establishing malaria surveillance posts along the border may facilitate early detection and treatment, helping to prevent localized onward transmission.

Previous studies have shown that individuals working in environments with livestock and animal farms face an increased risk of malaria due to conditions that promote vector proliferation [32–34]. Similarly, our study found that people engaged in livestock-related activities had a higher risk of *P. vivax* infection, even more so than agricultural workers, who are often considered high-risk due to their frequent exposure to forested areas [23]. However, malaria risk also depends on specific occupational activities. For example, agricultural workers who do not stay overnight in the fields may have a lower risk of infection. Nonetheless, implementing preventive measures, such as consistent bed net use whenever or wherever individuals sleep during dusk and dawn, remains crucial in reducing malaria transmission. Our findings also indicate that individuals working as government officers, typically in downtown office settings, and the unemployed, who primarily stay home to manage household responsibilities, had a lower risk of infection. This underscores the role of occupational exposure in malaria transmission. To complement personal protective measures, vector control strategies, including environmental manipulation and larval source reduction around livestock areas, should be implemented to mitigate malaria risk.

*Plasmodium vivax* malaria requires a complementary treatment to eliminate hypnozoite forms from the liver. In Thailand, a 14-day PQ regimen is implemented to achieve a radical cure, but it is administered without DOT [8]. Consequently, adherence to the full 14-day course remains challenging, as patients often discontinue treatment once symptoms subside [35]. Additional barriers to PQ use include contraindications in young children, pregnant and lactating women, and individuals with G6PD deficiency. A modeling study estimated that approximately 43% of *P. vivax* malaria cases result from relapses rather than new infections [36]. Although our study could not differentiate between relapsed and newly acquired infections, our findings indicate that individuals with a history of malaria had a higher likelihood of *P. vivax* infection, consistent with findings from other studies [23, 37]. Strengthening malaria prevention strategies to reduce new infections and promoting adherence to the 14-day PQ regimen are essential for effective malaria control.

Our study suggests that consistent bed net use serves as a protective factor against febrile *P. vivax* infection. Numerous studies have demonstrated the effectiveness of bed nets, including insecticide-treated nets (ITNs), in malaria prevention [37–39]. In Thailand, core vector control strategies include the distribution of LLINs and the implementation of indoor residual spraying (IRS) [8]. However, the uptake of LLINs among at-risk populations and the acceptance and feasibility of IRS remain suboptimal [10, 40]. Barriers to bed net use include occupational settings where using nets is impractical, as well as personal preferences such as concerns about skin allergies or discomfort in cold weather [10, 41]. Given the ongoing malaria transmission in these areas, even a single night of sleeping without a bed net increases the risk of acquiring an infection. Therefore, in addition to distributing ITNs, efforts should focus on promoting consistent, daily bed net use. The provision of forest-adapted bed nets, such as hammock nets, may also be considered to enhance protection for high-risk populations.

*Plasmodium vivax* malaria is generally less severe than *P. falciparum* malaria and typically presents with milder symptoms. This study, based on six years of longitudinal data, analyzed thousands of febrile *P. vivax* cases, making it, to our knowledge, the first of its kind. However, several limitations should be noted. First, the presence of fever among study participants may have resulted from coinfections with other illnesses in addition to malaria [42]. Although malaria diagnosis was performed using microscopy, other fever-related infections could not be entirely excluded, potentially leading to a slight overestimation of malaria-related fever prevalence. Second, the study was conducted in a border region adjacent to Myanmar, where large-scale migration has occurred, particularly since 2021. Consequently, the findings may not be generalizable to regions with different geographic and epidemiological contexts. Third, data were collected verbally through CRFs, which may not accurately reflect information such as malaria history and bed net usage due to recall bias or misreporting. Additionally, as the study utilized secondary data from a six-year longitudinal study, the availability and distribution of variables were beyond the researchers’ control. For example, the study sample predominantly consisted of children and students with no employment and individuals without reported travel or malaria history. This may have introduced selection bias and limited the generalizability of findings to other high-risk populations, such as working-age adults or individuals with frequent cross-border movement. Fourth, malaria diagnosis relied solely on microscopy, which, while more accurate than RDTs, is less sensitive than PCR testing, particularly for detecting low-density parasitemia [43]. Future studies could benefit from incorporating parasite quantification into the analysis. Lastly, the study utilized a PCD approach, meaning that malaria cases were identified only among individuals who sought care at health facilities. This method may have missed infections in those who did not seek medical attention, sought treatment at hospitals or clinics in other locations, or self-treated using pharmacies or alternative remedies.

## Conclusions

*Plasmodium vivax* malaria remains a significant public health concern in the GMS. This study identified a relatively high slide positivity rate of *P. vivax* infection in a malaria-endemic district in western Thailand along the Myanmar border. Sustaining and expanding malaria surveillance and treatment services in border areas are essential for effective disease control. Targeted interventions and health promotion strategies should focus on high-risk groups, particularly school-going children and working-age males with a history of malaria or recent travel. Additionally, promoting the consistent use of bed nets, ensuring adherence to the full course of hypnozoite-clearing treatment, and encouraging early malaria care-seeking and testing, especially among non-Thai citizens, will be crucial in reducing malaria transmission and improving disease outcomes.

## Data Availability

All data generated or analyzed during this study are included in the article. The de-identified raw dataset is available from the corresponding author upon reasonable request.

## Abbreviations

CRF: Case record form
DOT: Directly observed therapy
G6PD: Glucose-6-phosphate dehydrogenase
GMS: Greater Mekong Subregion
IRS: Indoor residual spraying
ITN: Insecticide-treated net
LLINs: Long-lasting insecticidal nets
PCD: Passive case detection
PCR: Polymerase chain reaction
RDT: Rapid diagnostic test
SEA: Southeast Asia
SPR: Slide positivity rate
